# Effect of SiC Nanoparticles on Hot Deformation Behavior and Processing Maps of Magnesium Alloy AZ91

**DOI:** 10.3390/nano8020082

**Published:** 2018-02-01

**Authors:** Kaibo Nie, Xinkai Kang, Kunkun Deng, Ting Wang, Yachao Guo, Hongxia Wang

**Affiliations:** 1 College of Materials Science and Engineering, Taiyuan University of Technology, Taiyuan 030024, China; xinkai.kang@hotmail.com (X.K.); dengkunkun@tyut.edu.cn (K.D.); wangt_123@hotmail.com (T.W.); guoychao@hotmail.com (Y.G.); wanghx_tut@hotmail.com (H.W.); 2 Shanxi Key Laboratory of Advanced Magnesium-Based Materials, Taiyuan University of Technology, Taiyuan 030024, China

**Keywords:** magnesium matrix nanocomposite, flow behavior, deformation mechanism, processing map

## Abstract

The hot deformation behavior and processing characteristics of AZ91 alloy and nano-SiCp/AZ91 composite were compared at temperature ranges of 523 K–673 K and strain rates of 0.001–1 s^−1^. Positive impact of SiC nanoparticles on pinning grain boundaries and inhibiting grain growth was not obvious when deformation temperature was below 623 K, but was remarkable when the temperature was above 623 K. By comparing compressive stress-strain curves of AZ91 alloy and nano-SiCp/AZ91 composites, the addition of nanoparticles could improve the deformation ability of a matrix alloy under high-temperature conditions. There was no essential difference of deformation mechanism between AZ91 alloy and the composite, but hot deformation activation energy of the composite was significantly lower than that of the AZ91 alloy. The AZ91 alloy and the composite had the same workability region of 600 K–673 K and 0.001–1 s^−1^, while instability region for the composite was reduced compared with that of AZ91 alloy at high temperature.

## 1. Introduction

With high demand for energy and fuel conservation in the transportation sector, there have been wide range of research interests focused on magnesium and magnesium-based alloys [1,2]. However, low strength, poor ductility and cold workability of magnesium alloy with hexagonal closed packed (HCP) structure and limited open slip systems hinder their applications. In general, mechanical properties of magnesium alloys can be improved with the addition of different alloying elements [3]. In spite of the enhanced properties, these are still inferior to basic engineering materials such as aluminum alloys and steel. Therefore, increasing attention has been paid to nanoparticle- or micro-particle-reinforced magnesium matrix composites, which exhibit higher mechanical properties at both ambient and high temperature, such as higher Young’s modulus, better creep resistance and friction resistance [4,5,6]. Various kinds of nano-ceramic particles such as carbides, nitrides, oxides as well as carbon nanotubes have been employed for the production of magnesium matrix composites [7,8,9,10]. Based on the author’s previous research, SiC nanoparticle-reinforced AZ91-based composite can be prepared by the combination of ultrasonic vibration and semisolid stirring [11]. This technology would help not only in adding ceramic nanoparticles but also in improving nanoparticles distribution.

The addition of brittle ceramic particles usually limits the plastic-forming ability of magnesium matrix composites at ambient temperatures. Thus, ceramic nanoparticle-reinforced magnesium matrix composite should be deformed at high temperatures, where additional slip systems of magnesium matrix can be activated [12]. Flow stress depending on deformation temperature, strain rate and deformation extent, can be used to describe the workability of magnesium matrix composite [13]. The hot deformation behaviors, kinetics of metallurgical transformation and processing maps for metal and its composite are of vital importance in conducting forming processes [14,15,16]. The constructive equations are frequently employed by investigators to understand the deformation mechanism while processing maps are the main technology to describe workability. Zhou et al. [14] studied hot deformation behavior and workability characteristics of bimodal size SiCp/AZ91 magnesium matrix composite with processing map, and found that characteristic microstructures predicted from the processing map were consistent with microstructure observations. According to processing maps, Srinivasan et al. [15] found that optimum conditions of both AZ31B magnesium alloy and AZ31 nanocomposite were at the deformation temperature of 400 °C and strain rate of 0.01. The processing map for Mg/nano-Al_2_O_3_ composite had been investigated by Prasad et al. [16]. It was found that optimum processing conditions were obtained at strain rates > 0.1 s^−1^ and temperature ranges of 400 °C–450 °C. Based on the above, in order to optimize hot deformation parameters, it is extremely significant and meaningful to study hot deformation behavior of magnesium matrix composite. Although a number of investigations have been made on the effect of nanoparticle addition to microstructures and mechanical properties of magnesium matrix [17,18,19], there are limited reports about the comparison of hot deformation behavior and processing maps between AZ91 magnesium alloy and nano-SiCp/AZ91 magnesium matrix composite, especially when prepared by semisolid stirring-assisted ultrasonic vibration.

Therefore, hot deformation behavior of AZ91 and nano-SiCp/AZ91 composite has been investigated by isothermal uniaxial compression under wide ranges of temperature and strain rate. Effects of SiC nanoparticles, deformation temperature and strain rate on the flow behaviors are analyzed. Based on dynamic material modeling (DMM), processing maps are constructed to optimize hot working domains of AZ91 and nano-SiCp/AZ91 composite. In addition, microstructure evolution with and without SiC nanoparticles is related to workability.

## 2. Materials and Methods

The matrix alloy in the present study is AZ91 magnesium alloy with a composition of Mg-9.07Al-0.68Zn-0.21Mn, and supplied by Northeast Light Alloy Company Limited, Longkou City, China. The reinforcement is SiC nanoparticle with an average particle size of 60 nm, and supplied by Hefei Kaier Nanometer Energy and Technology Company Limited, Hefei, China. The nano-SiCp/AZ91 composites (CN) have been prepared by the combination of ultrasonic vibration and semisolid stirring as described in detail in [11]. For the first step, AZ91 magnesium alloy was melted at 720 °C in an electrical resistance furnace with a shielding gas of CO_2_/SF_6_. For the second step, SiC nanoparticles with volume fraction of 1% were added through semisolid stirring when the melt was cooled to 590 °C. The stirring time was set to 5 min. After that the melt was reheated to 700 °C and subjected to ultrasonic vibration. The third step was the solidification of the melt in a preheated steel mold (450 °C) under pressure. 

Cylindrical specimens for compression test were machined from as-cast ingots. The diameter was 8 mm while the height was 12 mm for all the specimens. To minimize the effect of Mg_17_Al_12_ phase, solution treatment was carried out at 420 °C for 24 h before compression. Hot compression testing was conducted on a Gleeble 3500 test machine (Data Sciences International, New Brighton, MN, USA). Graphite was used as lubricant which can reduce the friction at the punch-specimen interface. Before compression, all specimens were conductively heated with heating rate of 5 K/s and kept at designed temperature for 5 min. So there was enough time to obtain a homogeneous temperature distribution throughout the specimen. After the strain reached 0.5, the compression was finished and all specimens were water quenched from test temperature. The deformation temperature ranged from 523 K to 673 K with an interval of 50 K while strain rate varied from 0.001 to 1 s^−1^ in the current work.

4XC optical microscope (OM) and MIRA 3XMU scanning electron microscope (SEM) were used for microstructure observation after hot compression. The specimens to be analyzed were cut parallel to compression direction using wire electrical discharge machining. The specimens were ground, polished and then etched [5 mL acetic acid + 6 g picric acid + 10 mL H_2_O + 100 mL ethanol (95%)].

## 3. Results and Discussion

### 3.1. Microstructure with and without Nano-SiCp after Hot Compression

**Figure 1:**
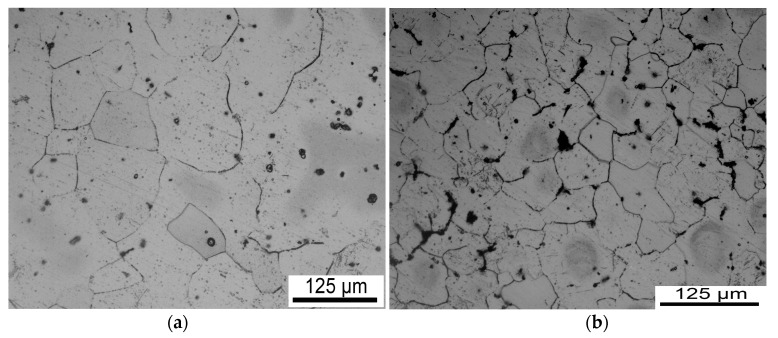
OM images of as-cast (**a**) AZ91 alloy (**b**) nano-SiCp/AZ91 composite.

**Figure 2:**
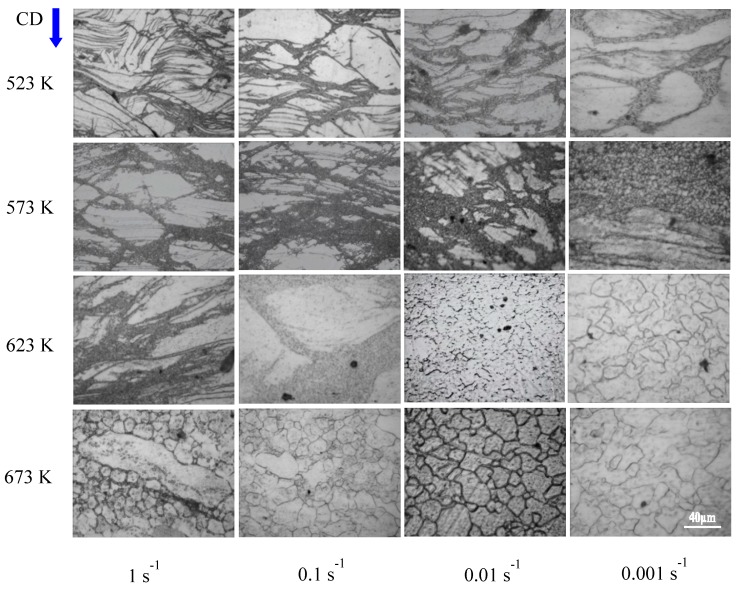
OM images of AZ91 alloy after hot compression. (CD represents compression direction).

Figure 1 shows OM images of AZ91 alloy and nano-SiCp/AZ91 composite in the as-cast condition. It can be seen that with the addition of SiC nanoparticle matrix grains of the composite is significantly refined compared with that of the AZ91 as shown in Figure 1a,b. Figure 2 shows OM images of as-cast AZ91 alloy after hot compression under different deformation conditions. Under the same deformation temperature, the extent of dynamic recrystallization (DRX) increases with decreasing strain rate from 1 s^−1^ to 0.001 s^−1^. With the increase of deformation temperature from 523 K to 673 K, DRX extent also increases under the same strain rate. There are a high amount of twins existing in the AZ91 at a deformation temperature of 523 K and strain rates of 1 s^−1^ and 0.1 s^−1^. The number of enabled slip systems is few for magnesium with HCP structure, and larger critical shear stress is required for the activation of basal slip system in low temperature and high speed [20]. Thus, slip system is difficult to open, and twinning induced by stress concentration is the main deformation way for the present alloy. When deformation temperature is 673 K, dynamic recrystallization with volume fraction of 50% is formed in the alloy at high strain rate of 1 s^−1^. The microstructure is made up of fine recrystallized grains and a few coarse grains. The volume fraction of dynamic recrystallization is 98% at a deformation temperature of 673 K and a strain rate of 0.01 s^−1^. Further decreasing the strain rate to 0.001 s^−1^, fine recrystallized grains grow up and the deformed structure tends to coarsen. 

**Figure 3:**
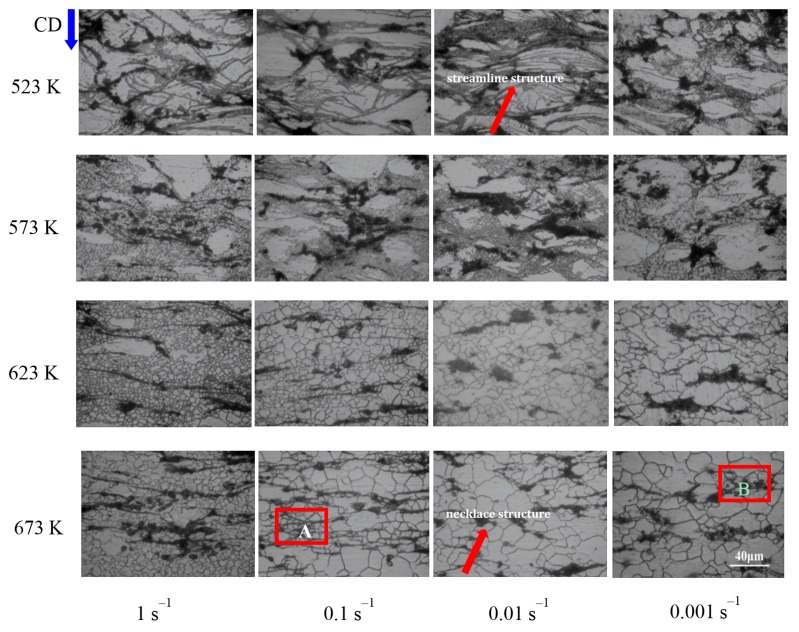
OM images of nano-SiCp/AZ91 composite after hot compression. (CD represents compression direction, red arrows indicate necklace structure; rectangular red areas “A” and “B”represent the grains away from SiC nanoparticles).

With respect to nano-SiCp/AZ91 composite, as shown in Figure 3, necklace structure (as indicated by the red arrows) distributed perpendicular to compression direction becomes more and more obvious. With the accumulation of strain, radial force perpendicular to the compression direction increases, leading to elongated grains along the radial direction. Meanwhile, matrix softening appears in the matrix alloy with the increase of deformation temperature. Thus, particle mobility is improved and SiC nanoparticles can flow with the matrix alloy, resulting in the appearance of necklace structure distributed perpendicular to the compression direction. It can be also found that recrystallization extent is enhanced with increasing the deformation temperature. When the deformation temperatures are 523 K and 573 K, volume fraction of DRX is low and recrystallized grains are fine. There are no obvious differences for the distribution of recrystallized grains around and away from SiC nanoparticles at this situation. While the deformation temperature is 623 K and the strain rate is 1 s^−1^, volume fraction of DRX reaches 80% in the matrix alloy. Among the four deformation temperatures, microstructure deformed at 623 K is relatively uniform under different strain rates. As deformation temperature further increases to 673 K, complete recrystallization and grain coarsening take place. The grain size is small around SiC nanoparticles while the grains are coarse away from SiC nanoparticles within the rectangular red areas “A” and “B” at 673 K as shown in Figure 2. In the present work, a pinning effect by the addition of only 1 vol. % SiC nanoparticles can inhibit the growth of recrystallized grain during the hot compression. As a result, grain size of the nano-SiCp/AZ91 composite tends to be bimodal. Under the same deformation temperature there is slight increase in the recrystallization extent with decreasing the strain rate. Thus, deformation temperature plays a more significant role in microstructure change as compared to the strain rate. The volume fraction of DRX increases while the number of twins decreases with the decrease of strain rate at low temperatures of 523 K and 573 K. The number of precipitated phases increases because there is sufficient time for solute atoms to migrate at low temperature and slow strain rate. In contrast, the recrystallization extent of the matrix is full and recrystallized grains grow up with the decrease of strain rate at 623 K and 673 K.

**Figure 4:**
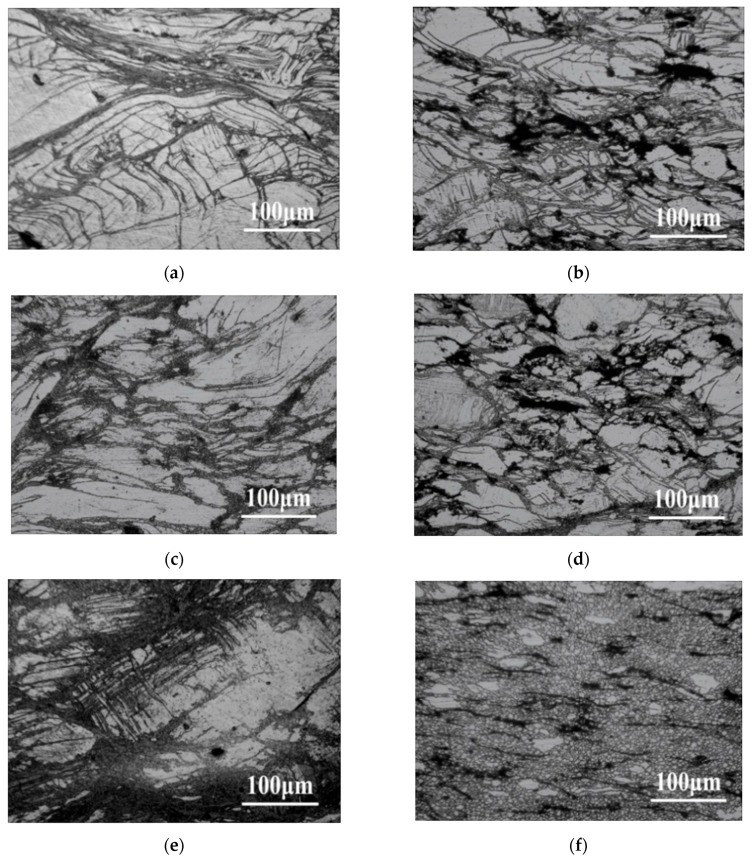

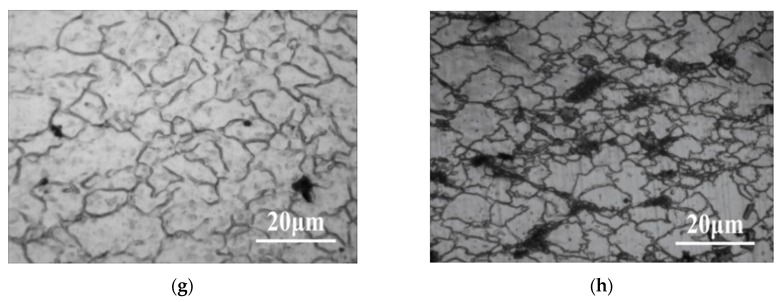
OM images of (**a**,**c**,**e**,**g**) AZ91 alloy, (**b**,**d**,**f**,**h**) composite after hot compression: (**a**,**b**) 523 K, 1 s^−1^; (**c**,**d**) 523 K, 0.01 s^−1^; (**e**,**f**) 623 K, 1 s^−1^; (**g**,**h**) 623 K, 0.001 s^−1^.

The comparison of OM images of AZ91 and nano-SiCp/AZ91 composite is given in Figure 4. There is no obvious difference in the microstructures between AZ91 and nano-SiCp/AZ91 composite at a deformation temperature of 523 K as shown in Figure 4a–d, which are made up of twins and streamline structure. However, under the deformation condition of 623 K/1 s^−1^, as shown in Figure 4e,f, microstructure of AZ91 alloy is composed of some recrystallization grains with streamline structure, a large number of twins and initial microstructure. By contrast, a large number of recrystallized grains are formed in the composite with no twins and streamline structure. This can be attributed to the addition of nanoparticles as well as the different initial microstructure of the matrix. The kinetics of dynamic recrystallization were expected to be accelerated by a decrease in grain size [21]. Since the initial grain size of the composite is obviously decreased compared with that of the AZ91 as shown in Figure 1, the AZ91 with coarser grains displays a mixed structure and the composite with finer grains shows equiaxed grain structure. When the strain rate decreases to 0.001 s^−1^ at 623 K, original grains in either AZ91 or composite are completely replaced by recrystallized grains, and recrystallized grain coarsening occurs. The average grain size of AZ91 alloy is 25.4 μm while that of the composite is 20.1 μm at deformation condition of 623 K/0.001 s^−1^, indicating the addition of SiC nanoparticles can refine the matrix alloy. Accordingly, the effect of SiC nanoparticles on the DRX changes with deformation temperature. When the deformation temperature is below 623 K, the positive impact of SiC nanoparticles on the DRX is not obvious but remarkable when the temperature is above 623 K. As mentioned earlier, during low-temperature compression, slip systems of AZ91 and nano-SiCp/AZ91 composite are limited and difficult to activate. So basal slip accompanied by twinning (giving c+a strain) is the main way for incoordinate deformation. With the increase of deformation temperature, the mobility of dislocation increase and more slip systems can be activated. Thus, the c+a glide mechanism becomes the main way and twinning ceases this role at high-temperature compression. The dislocation source can produce a large number of dislocations at high strain rate, which cannot bypass the hard SiC nanoparticles distributed evenly in the matrix alloy and form a dislocation tangle. When the number of dislocations pinned by SiC nanoparticles reaches a certain level, tangled dislocations generate a mass of dislocation cells. As the surrounding stress field increases, dislocation cells develop into unstable sub-structures. These sub-structures can further form low-angle grain boundaries and, finally, recrystallized grains. Correspondingly, at high deformation temperature the matrix alloy is softened and a pinning effect of the SiC nanoparticles on the dislocations is weakened. In this situation, the SiC nanoparticles play a role in pinning grain boundaries and inhibiting grain growth.

**Figure 5:**
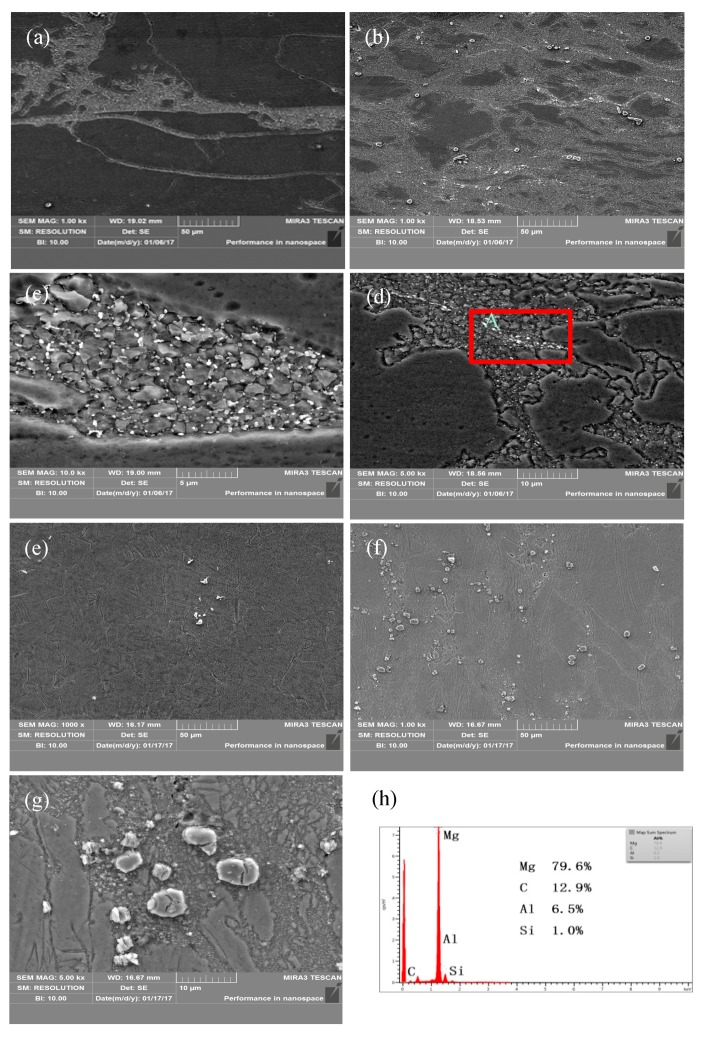
SEM images of (**a**,**c**,**e**) AZ91 alloy, (**b**,**d**,**f**,**g**,**h**) composite after hot compression: (**a**,**b**) 573 K, 0.01 s^−1^; (**e**,**f**) 673 K, 0.001 s^−1^; (**c**,**d**) the high magnification of (**a**,**b**) respectively; (**g**) the high magnification of (**f**); (**h**) the EDS of area “A” in (**d**). (Red rectangular frame “A” indicates the region of EDS analysis)

Figure 5 shows SEM microstructures of AZ91 alloy and nano-SiCp/AZ91 composite. As shown in Figure 5a,b, there are more precipitated phases in the composite than that of AZ91 alloy under the same compression conditions. Research indicates that the stress state of magnesium alloy is not stable during deformation and crystal rotates with the change of stress state, which finally forms the region with stable stress orientation [22]. This region is known as the deformation zone where Mg_17_Al_12_ phases tend to precipitate preferentially in the form of fine particles. In this current work, precipitated spherical particles are mainly distributed in the deformed microstructure of AZ91 alloy. With respect to nano-SiCp/AZ91 composite, Mg_17_Al_12_ phases mainly exist in three forms: dispersed block, fine spherical particles and streamline shape. It should be noted that a large number of spherical second phases precipitate along the recrystallized grain boundaries both in the AZ91 alloy and nano-SiCp/AZ91 composite in Figure 5c,d. This phenomenon is mainly due to high density of dislocation and defects on the DRX grain boundaries, which provides favorable nucleation energy and nucleation sites for the precipitates. Besides, since the addition of SiC nanoparticles can promote the DRX of matrix alloy, grain boundaries of recrystallized grains can provide more nucleation sites, which facilitates the precipitation of Mg_17_Al_12_ phases. The strain increases continuously and the stress field increases gradually during the precipitation of Mg_17_Al_12_ phases [23]. When the accumulated strain and stress reach a certain extent, the matrix alloy can be induced to recover and form recrystallized grains. DRX induced by strain, stress and stress field occurs preferentially on the grain boundaries during the precipitation process. The recrystallized grain boundaries provide the nucleation sites for the Mg_17_Al_12_ phases, while the precipitation of Mg_17_Al_12_ phases can promote DRX, and the two aspects are mutually promotive. EDS analysis of the region “A” with red rectangular frame in Figure 5d, dispersed SiC nanoparticles exist as shown in Figure 5h. At a high temperature of 673 K, it can be still observed that the amount of precipitated phases in the composite is larger than that of the alloy as shown in Figure 5e,f. Under the high-temperature compression, SiC nanoparticles can flow with the matrix alloy and form a streamline shape. The SiC nanoparticles with high hardness dispersed in AZ91 matrix can only be bypassed by dislocation. This pinning effect leads to a dislocation tangle, which provides a channel for the diffusion and migration of solute atoms. Thus, supersaturated solid solution would form and Mg_17_Al_12_ phases precipitate in this area. However, due to the low content of SiC nanoparticles (1 vol. %) and slow strain rate (0.01 s^−1^) the number of dislocation tangles is low, and size as well as amount of precipitated phases in the form of streamline shape is low. In Figure 5g under deformation condition of 673 K/0.001 s^−1^, it can be found that the Mg_17_Al_12_ phases in block form are distributed within the recrystallized area surrounding by fine Mg_17_Al_12_ phases. At high-temperature deformation, magnesium alloy can coordinate deformation by grain boundary sliding [24]. After high-temperature compression, grain size of fine-equiaxed DRX is below 10 μm, which has the ability of grain boundary sliding. The grain rotation and flow can be induced by compressive stress, resulting in large shear stress. Under shear stress the massive Mg_17_Al_12_ phases are broken and flow with the grain boundary sliding at slow strain rate and high deformation temperature.

### 3.2. Compressive True Stress-True Strain Curves with and without Nano-SiCp

**Figure 6:**
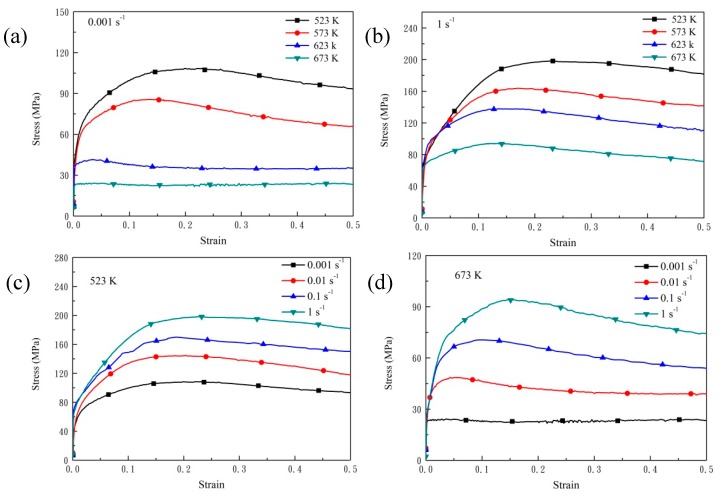
Compressive true stress-strain curves of AZ91 alloy: (**a**) 0.001 s^−1^; (**b**) 1 s^−1^; (**c**) 523 K; (**d**) 673 K.

Figure 6 shows compressive-true stress-true strain curves of AZ91 alloy under different compression conditions. As the strain increases, the flow stress first increases, then decreases, and finally becomes stable. In general, a typical stress-strain curve consists of four stages: work hardening, transition, softening and steady. In addition, work hardening and dynamic softening coexist and compete with each other. In the initial stage of compression, elastic deformation occurs and flow stress increases linearly when the strain increases slightly. After that, as the strain increases, the flow stress increases slowly and reaches the peak stress of the whole compression curve, at which stage work hardening is dominant. When the strain further increases, flow stress decreases because the strengthening effect caused by work hardening is weakened by the softening effect of DRX. Finally, a steady state is achieved due to a balance between work hardening and dynamic softening. The flow stress of AZ91 alloy decreases with the increase of deformation temperature when strain rate is 0.001 s^−1^ as shown in Figure 6a. The non-basal slip system of AZ91 can be activated with relative low shear stress at high temperature, which decreases the deformation resistance. The increased mobility of dislocations at elevated temperatures reduces dislocation tangle and impairs work hardening. Moreover, volume fraction of DRX was obviously improved at high temperatures and softening effect by DRX was significantly enhanced. As shown in Figure 6c, flow stress of AZ91 alloy decreases with decreasing strain rate at 523 K. The extent of recrystallization at high strain rate is not full under a short deformation time. Thus, the softening effect caused by recrystallization is delayed while work hardening resulting from a large number of dislocations tangle increases.

**Figure 7:**
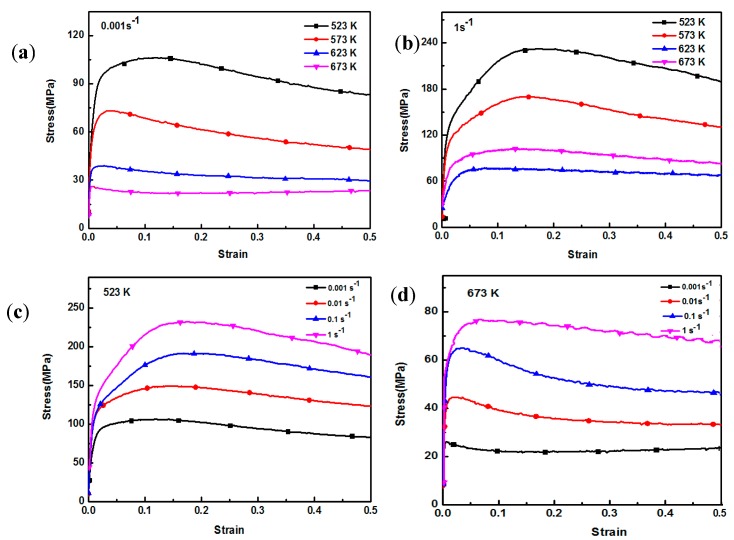
Compressive true stress-strain curves of nano-SiCp/AZ91 composite: (**a**) 0.001 s^−1^; (**b**) 1 s^−1^; (**c**) 523 K; (**d**) 673 K.

Figure 7 gives compressive-true stress-true strain curves of nano-SiCp/AZ91 composite under different compression conditions. The change trend in the relationship between flow stress and strain for the composite is similar to AZ91 alloy. As shown in Figure 7a,b, under the same strain rates there are four stages on the stress-strain curve when deformation temperature is below 573 K. The flow stresses below 573 K are significantly higher than those above 623 K. When the deformation temperature is above 623 K, steady stage of the stress-strain curve is prolonged and the flow stress increases slowly with the increase of strain. At high temperatures full recrystallization of matrix alloy in the composite is achieved in a short time (Figure 3) and then the recrystallized grains coarsen, so the mobility of grain boundaries increases. The stress concentration from the non-uniform deformation between neighboring grains is reduced and the inhabitation of SiC nanoparticles on the dislocations is weakened. Due to the above factors the generation and annihilation of dislocations reach a dynamic equilibrium, and a balance between softening and hardening is achieved. In Figure 7a the stress-strain curve shows an obvious work-hardening stage at 523 K while not evident at other three deformation temperatures (573 K, 623 K and 673 K). As shown in Figure 7c, the work hardening stage is extended with the increase of strain rate at a temperature of 523 K. After peak stress, the curve slows down and the softening stage lengthens. This is because at low deformation temperatures and high strain rates twinning is the main way to coordinate deformation for nano-SiCp/AZ91 composite. Besides, volume fraction of DXR is low in the composite, resulting in a weak softening effect. At the same time, it is difficult to coordinate the plastic deformation between the nanoparticles and the matrix in a short time, which can prolong the hardening stage and enhance peak stress. The flow stress reaches the peak stress with slight strain and then decreases significantly with increasing strain at 673 K, as shown in Figure 7d. The incubation period of recrystallization is reduced at a high deformation temperature. Thus, DRX can occur under slight strain, resulting in a significant softening effect and the lowering of the stress-strain curve. 

By comparing compressive stress-strain curves of AZ91 alloy and nano-SiCp/AZ91 composites, the addition of nanoparticles can improve the deformation ability of the matrix alloy under high temperature conditions. The flow stress of AZ91 alloy at 673 K is larger than that of the composite when the strain rate is above 0.1 s^−1^. The difference of flow stresses between AZ91 and its composite is decreased when strain rate is below 0.1 s^−1^. Table 1 shows grain sizes of AZ91 alloy and nano-SiCp/AZ91 composite under different deformation conditions. It can be seen from Table 1 that grain size of the composite with full recrystallization is smaller than that of the AZ91. When the strain rate is above 0.1 s^−1^ at 673 K, the matrix has completed a greater extent of recrystallization compared with that of the AZ91 with a large amount of primary coarse grains (as shown in Figure 2 and Figure 3). Initial fine-grained material is expected to DRX faster (and therefore soften more quickly) than initial coarse-grained material due to higher numbers of grain boundary DRX nuclei, giving rise to lower deformation stresses, an effect opposite in sense to that predicted by the Hall-Petch law [25]. Thus, for the composite strengthening effect of SiC nanoparticles is lower than the softening effect of grain refinement, resulting in the lower peak stress and flow stress in the steady state on the stress-strain curves compared with that of AZ91. However, when the strain rate is below 0.1 s^−1^, a large degree of DRX can be found in both AZ91 alloy and nano-SiCp/AZ91 composite. Because SiC nanoparticles can promote the precipitation of Mg_17_Al_12_ and refine grains, the amount of precipitates is larger while the grain size of the composite is smaller than that of AZ91 alloy. The competition between softening effect of Mg_17_Al_12_ at high temperature and reinforcing effect of SiC nanoparticles leads to inconspicuous differences of flow stress between AZ91 alloy and nano-SiCp/AZ91 composite. 

**Figure 8:**
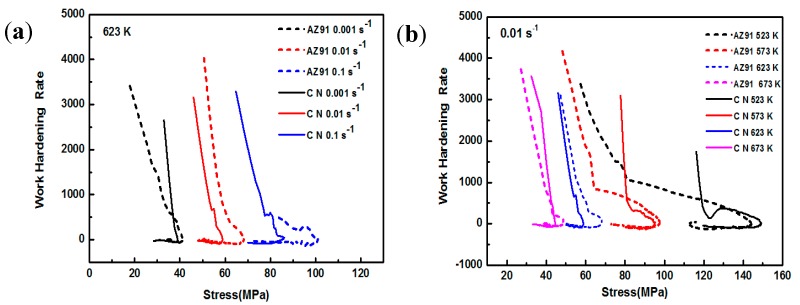
Schematic charts θ-σ of AZ91 alloy and nano-SiCp/AZ91 composite: (**a**) 623 K, (**b**) 0. 01 s^−1^.

**Figure 9:**
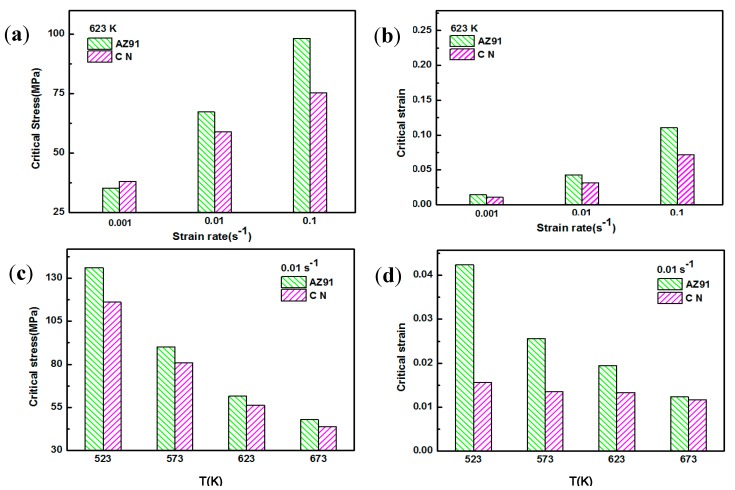
The critical stress and strain of AZ91 and nano-SiCp/AZ91 composite: (**a**,**b**) 623 K, (**c**,**d**) 0.01 s^−1^.

Based on the above phenomenon, deformation temperature and strain rate have an important effect on the deformation behavior for both AZ91 alloy and nano-SiCp/AZ91 composite. During hot deformation the appearance of peak stress in the micro-SiCp reinforced magnesium matrix composites indicates the occurrence of DRX [26]. When the strain accumulates to a certain extent, DRX can be induced and volume fraction of DRX increases with the further increase of the strain. At the same time, the stress-strain curve rises to the peak value and then decreases with the increase of strain. Maksoud et al. [27] found that during hot deformation of AZ31 alloy the occurrence of peak stress was the only criterion for recrystallization, and DRX had taken place before the peak stress. McQueen et al. [28] suggested that DRX existed in the material although there was no obvious peak stress in the stress-strain curve, which further indicate DRX has taken place before the peak strain is reached. Thus, critical strain of recrystallization is pivotal to control deformed microstructures. According to the theory of work hardening, the variation of stress *δ* and work hardening rate *θ* can be divided into five stages [28]: sliding, work-hardening, dynamic recovery, strain hardening and finally the dislocation density reaches the critical value required for recrystallization as the strain accumulates to a certain extent. In this case, the *θ*-*σ* curve dives downward and the stress corresponding to the inflection point is the peak stress (*σ*_p_) [29]. Poliak et al. [30] gave the (d*θ*/d*σ*)/*σ* curve based on the *θ*-*σ* curve, and the stress at the lowest point on the curve is the critical stress. Then the critical strain is determined on the stress-strain curve according to the critical stress. Therefore, by applying work hardening rate, critical stresses of AZ91 alloy and nano-SiCp/AZ91 composite under different compression conditions can be judged according to the critical stress pattern of magnesium alloy [31]. Figure 8 shows the relationship between *θ* and *σ* for AZ91 alloy and nano-SiCp/AZ91 composite under different compression conditions. In order to investigate the effect of SiC nanoparticles on critical condition of recrystallization, critical strain of AZ91 alloy and its composite during hot compression is compared in this work as shown in Figure 9. Figure 9a,c shows critical stresses of AZ91 alloy and nano-SiCp/AZ91 composite under different compression conditions. The critical strain can be determined according to the critical stress as shown in Figure 9b,d. The critical strain increases with the increase of strain rate and the decrease of deformation temperature for both AZ91 alloy and its composite. Compared with the AZ91 the composite has a lower critical strain and reaches its critical strain earlier. This is due to the fact that the nanoparticles can provide favorable conditions for recrystallization nucleation and refine the grains. The content of SiC nanoparticles is low along the grain boundaries and the grain boundaries without nanoparticles can still bow out to form recrystallized grains. As a result, the pinning effect of the nanoparticles on dislocations movement provides favorable conditions for recrystallization nucleation. 

### 3.3. Constitutive Analysis with and without Nano-SiCp

The relationship between strain rate and flow stress during thermal deformation can be described by constitutive equations [32]. There are three typical constitutive equations, that is: power, exponential and hyperbolic constitutive. In general, power law can be used at low stress, exponential law was applied at high stress and hyperbolic sine law was suitable for a wider stress range. Therefore, in the present work, the hyperbolic sine law was adopted to describe the deformation behavior of AZ91 alloy and nano-SiCp/AZ91 composite, and the stress value was determined with the strain of 0.5. 

Three typical constitutive equations are shown in Equations (1)–(3) [33]:

(1) $$\dot{\varepsilon }=A_{1}\sigma^{n_{1}}\exp\left(-\frac{Q}{RT}\right)$$

(2) $$\dot{\varepsilon }=A_{2}\exp\left(\beta \sigma \right)\exp\left(-\frac{Q}{RT}\right)$$

(3) $$\dot{\varepsilon }=A_{3}{\left(\sinh(\alpha \sigma )\right)}^{n}\exp\left(-\frac{Q}{RT}\right)$$

where *A*_1_, *A*_2_, *A*_3_, *α* and *β* are material constant, $\dot{\varepsilon }$ is strain rate, *δ* is flow stress, *n*_1_ and *n* are stress exponents, *Q* is the activation energy during hot deformation, *R* is gas constant (8.31 J mol^−1^ K^−1^) and *T* is absolute temperature. The calculation of activation energy and stress exponent can be used to determine the deformation mechanism at high temperature. 

The effects of the strain rate $\dot{\varepsilon }$ and temperature *T* can be combined by the Zener–Hollomon parameter Z as given in Equation (4):

(4) $$Z=\dot{\varepsilon }\exp(\frac{Q}{RT})$$

Taking natural logarithm for both sides of Equation (1), it can be expressed by:

(5) $$\ln\dot{\varepsilon }=n_{1}\ln\sigma +\ln A_{1}-\frac{Q}{RT}$$

**Figure 10:**
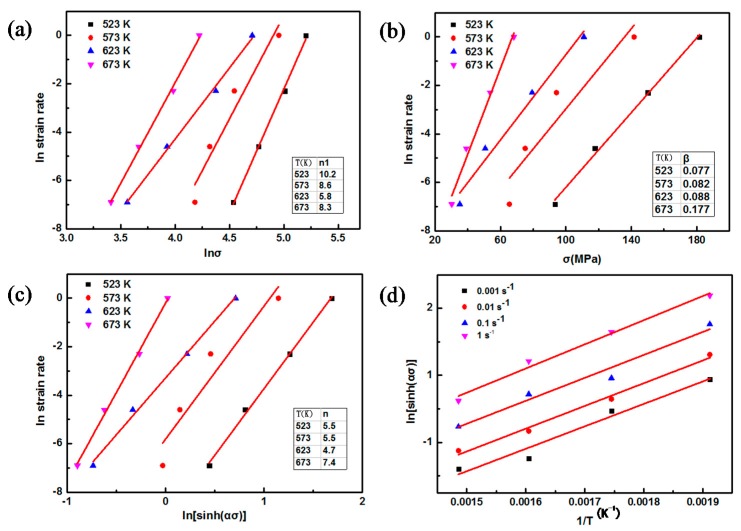
Linear fitting of AZ91 alloy (**a**) $\ln\dot{\varepsilon }$ − ln*σ*; (**b**) $\ln\dot{\varepsilon }$ − *σ*; (**c**) $\dot{\varepsilon }$ − ln(sinh(*ασ*)); (**d**) ln(sinh (*ασ*)) − 1/*T*.

The value of stress exponent *n*_1_ can be obtained by the plot of $\ln\dot{\varepsilon }-\ln\sigma$, as shown in Figure 10a.

Taking natural logarithm for both sides of Equation (2), it can be expressed by:

(6) $$\ln\dot{\varepsilon }=\sigma \beta +\ln A_{2}-\frac{Q}{RT}$$

The value of material constant *β* can be obtained by fitting the curve of $\ln\dot{\varepsilon }-\sigma$ as shown in Figure 10b. It can be seen from Figure 10 that the values of *n*_1_ and *β* are 8.0 and 0.10532, respectively. Thus, the value of *α* can be determined according to formula *α* = *n*_1_/*β*. 

Taking natural logarithm for both sides of Equation (3), it can be expressed by:

(7) $$\ln\dot{\varepsilon }=n\ln\left(\sinh(\alpha \sigma )\right)+\ln A_{3}-\frac{Q}{RT}$$

By substituting the value of α into Equation (7), the value of stress exponent *n* can be obtained by fitting the curve of $\ln\dot{\varepsilon }-\ln\left(\sinh(\alpha \sigma )\right)$ as given in Figure 10c. 

Equation (8) is obtained based on Equation (7), which can be expressed:

(8) $$\ln\left(\sinh(\alpha \sigma )\right)=\frac{Q}{nRT}+\frac{\ln\dot{\varepsilon }}{n}-\frac{\ln A_{3}}{n}$$

Linear regression analysis was performed on the curve of ln(sinh(ασ))−1/T as shown in Figure 10d. The stress exponent *n* for AZ91 alloy can be obtained by linear regression analysis of Figure 10c, as shown in Table 2.

According to Equation (3), the activation energy *Q* can be described by Equation (9):

(9) $$Q=R(\frac{\partial \ln\left(\sinh(\alpha \sigma )\right)}{\partial \left(1/T\right)})\dot{\varepsilon }\;(\frac{\partial \ln\overset{\cdot }{\varepsilon }}{\partial \ln\left(\sinh(\alpha \sigma )\right)})T$$

As shown in Table 3, the activation energy can be obtained based on the linear regression analysis of Equations (3) and (7), and Figure 10c,d. It was reported that the value change of *n* is related to different deformation mechanism [34]: *n* = 2 for grain boundary glide, *n* = 3 for dislocation glide, *n* = 5 for dislocation climb and *n* = 8 for constant substructure model. When the deformation temperature ranges from 523 K to 623 K, the value of activation energy *Q* is close to the activation energy of lattice diffusion (*Q*_L_ = 135 kJ/mol) [35]. The value of stress exponent *n* under these compression conditions is close to 5 and the deformation mechanism can be determined as the dislocation climbing mechanism controlled by lattice diffusion. When the deformation temperature is 673 K, the value of activation energy *Q* is higher than 200 kJ/mol. Due to the high deformation temperature recrystallization extent of the AZ91 alloy is high even at high strain rate, and the value of stress exponent *n* is 7.4 at 673 K (Figure 10c). Thus, the deformation mechanism under this situation is the constant substructure model.

**Figure 11:**
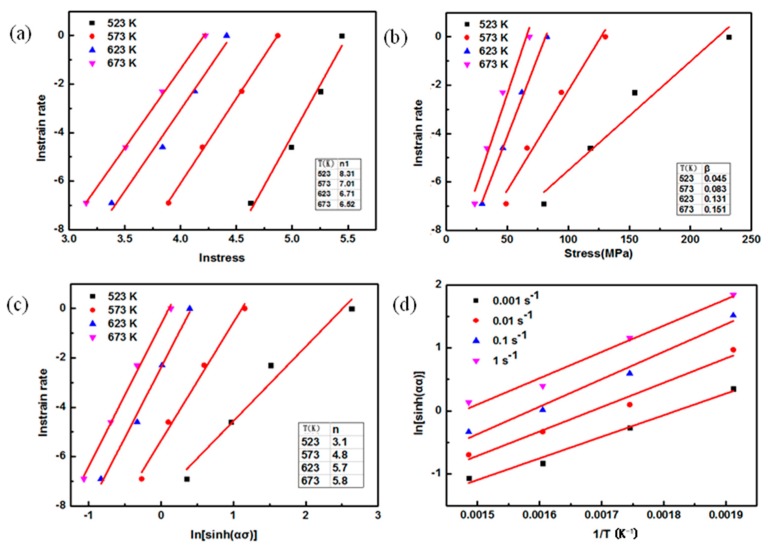
Linear fitting of nano-SiCp/AZ91 composite: (**a**) $\ln\dot{\varepsilon }$ − ln*σ*; (**b**) $\ln\dot{\varepsilon }$ − σ; (**c**) $\dot{\varepsilon }$ − ln (sinh(*ασ*)); (**d**) ln(sinh(*ασ*)) − 1/*T*.

With respect to nano-SiCp/AZ91 composite, as shown in Figure 11, the plots of $\ln\dot{\varepsilon }-\ln\sigma$, $\ln\dot{\varepsilon }-\sigma$, $\ln\dot{\varepsilon }-\ln\left(\sinh(\alpha \sigma )\right)$ and ln(sinh(ασ))−1/T can be obtained based on the analysis of Equations (5)–(8). The stress exponent *n* for the composite can be obtained by linear regression analysis of Figure 11c as shown in Table 4. As shown in Table 5, the activation energy *Q* for the composite can be described based on the linear regression analysis of Equations (8) and (9), and Figure 11c,d. In Table 4, it is found that the value of stress exponent for the composite increases with the increase of deformation temperature. With increasing the deformation temperature and strain rate, activation energy for the composite increases as shown in Table 5. When the deformation temperature is 523 K, the value of activation energy *Q* for the composite ranges from 86 to 104.74 kJ/mol, which is close to the activation energy of grain boundary diffusion (*Q*_gb_ = 82–105 kJ/mol) for pure magnesium [36]. The value of stress exponent *n* at 523 K is 3.1, so deformation mechanism for the composite at this situation can be determined as dislocation glide. When the deformation temperature varies from 573 K to 673 K, the value of activation energy *Q* is larger than the activation energy of lattice diffusion (*Q*_L_ = 135 kJ/mol) [36]. This indicates that the deformation behavior for the nano-SiCp/AZ91 composite is not common lattice self-diffusion. Due to the high volume fraction of DRX at high temperature, non-basal plane slip can be activated in the magnesium alloy. At temperature interval of 573–673 K, the value of *n* for the composite is 4–6. Thus, the deformation mechanism of nano-SiCp/AZ91 composite is controlled by dislocation climb resulting from lattice diffusion. 

**Figure 12:**
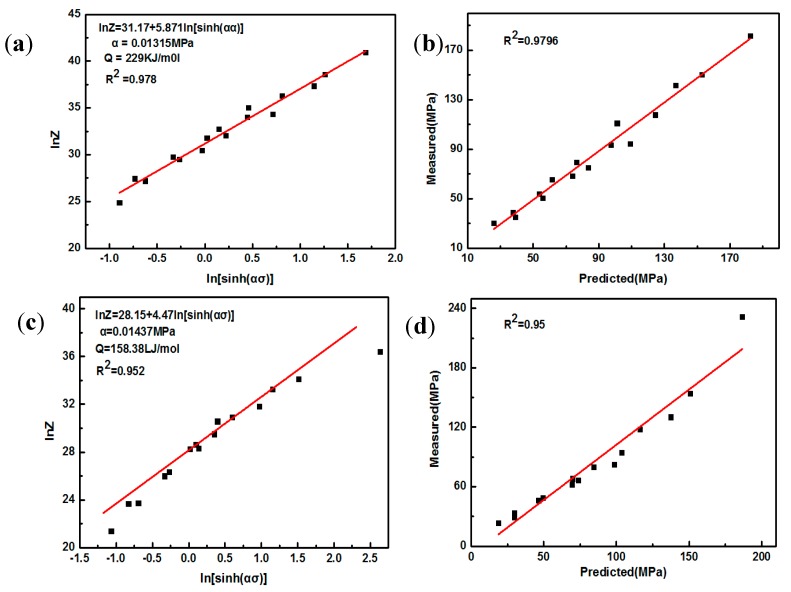
Linear fitting of (**a**) ln*Z* − (*sinh*(*ασ*)); (**b**) theoretical-actual stress of AZ91; (**c**) ln*Z* − *l*(*sinh*(*ασ*)); (**d**) theoretical- actual stress of nano-SiCp/AZ91 composite.

By substituting activation energy *Q* at different deformation condition (Table 3 and Table 5) into Equation (4), as shown in Figure 12a,c, value of A and constitutive equation of parameters can be evaluated using linear fitting curves of lnZ − *ln*(*sin*h(*ασ*)) according to Equations (3) and (4). Actual measurement and calculated value of flow stress are illustrated and fitted, as shown in Figure 12b,d, and linearly dependent coefficient R between measured value and calculated value is close to 1 for both AZ91 alloy and composite. This high fitting degree shows that hyperbolic sine can be applied to describe AZ91 alloy and composite under the present work. As shown in Table 6, by comparing the value of *n* it is found that the dominant deformation mechanism is the same for both AZ91 alloy and composite, and the hot activation energy of the composite is significantly lower than that of the AZ91 alloy. This phenomenon can be attributed to the following reasons. Firstly, with the addition of SiC nanoparticle original microstructure of nano-SiCp/AZ91 composite is finer than that of AZ91 alloy. Secondly, SiC nanoparticles can hinder grain boundary migration during hot compression, leading to refined grains of the composite and reduced deformation resistance. Based on the above analysis, since the addition of SiC nanoparticles can reduce the critical stress and critical strain of the composite, recrystallization will occur more easily. In addition, it is reported that after adding SiC nanoparticles the nonuniformity of deformation in the matrix alloy increases with the increase of strain during the deformation process [37]. The nonuniform deformed region for the composite is larger than that of AZ91 alloy, which has a great influence on the deformation mechanism because induced stress field resulting from non-uniform deformation can affect the dislocation motion.

### 3.4. Processing Maps with and without Nano-SiCp

In a hot-working process, the relationship between flow stress *σ* and strain rate $\dot{\varepsilon }$ can be represented by Equation (10) [38].

(10) $$\sigma =K{\dot{\varepsilon }}^{m}$$

The curve of $\ln\sigma -\ln\overset{\cdot }{\varepsilon }$ was fitted by cubic spline function and the accuracy of the *m* value is guaranteed by the coefficient. The power dissipation factor at the same temperature is represented by Equation (11) [39].

(11) $$m=\ln\sigma /\ln\overset{\cdot }{\varepsilon }$$

During the DMM the property for power dissipation due to microstructures evolution can be expressed by the dissipative efficiency of power [39], which can be expressed as Equation (12):

(12) $$\eta =\frac{2m}{2m+1}$$

where η is defined as proportional relation between energy consumed by the microstructures change and linear dissipative energy during deformation. The value of *m* is variable. The microstructure during the material processing can be controlled according to the *m* value. The power dissipation map in a two-dimensional plane can be obtained using deformation temperature and strain rate, which is generally described as an iso-efficiency counter map. Equation (13) shows the criterion used to define the onset of flow instability [39]:

(13) $$\zeta \left(\dot{\varepsilon }\right)=\frac{\partial \ln\left(\frac{m}{m+1}\right)}{\partial \ln\varepsilon \dot{\varepsilon }}+m\leq 0$$

where $\zeta \left(\dot{\varepsilon }\right)$ is instability parameter that is related to strain rate and deformation temperature. When $\zeta \left(\dot{\varepsilon }\right)$ value is positive, steady state appears for the deformation processing. When the $\zeta \left(\dot{\varepsilon }\right)$ value is negative, flow instability occurs. The variation of instability parameter represents an instability map, which defines the regimes of flow instability.

**Figure 13:**
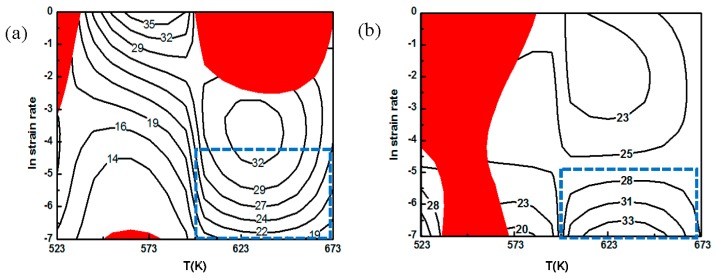
Processing maps of the AZ91 alloy and nano-SiCp/AZ91 composite at a strain of 0.5: (**a**) AZ91; (**b**) nano-SiCp/AZ91 composite [12].

Figure 13 shows processing maps of AZ91 alloy and nano-SiCp/AZ91 composite at a strain of 0.5. The red shaded area represents instability areas and the number of the equivalent line indicates the value of power dissipation efficiency (%). For the AZ91 alloy in Figure 13a, when the strain is 0.5, power dissipation efficiency increases and the values are located in the range of 30–35%. The instability region appears at a temperature ranges of 523 K–573 K and strain rate of 1 s^−1^. Ideal workability region of AZ91 alloy, marked by blue rectangle, corresponds to the processing conditions of 600 K–673 K/0.001–0.01 s^−1^. In this area, the power dissipation efficiency is large and no flow instability occurs. This can be related to stable microstructure and good mechanical properties of AZ91 alloy after hot deformation. The processing map for nano-SiCp/AZ91 composite is shown in Figure 13b, which is redraw based on our previous work [12]. The red shadow area in the nano-SiCp/AZ91 composite is mainly distributed at low temperature (573 K). This flow instability of the composite occurs at strain rate of 0.001–1 s^−1^ and instability area is large at low temperature, which indicates that the present composites are not suitable for thermal deformation at low temperature. The ideal workability region of the composite marked by blue rectangle corresponds to the processing conditions of 600 K–673 K/0.001–0.01 s^−1^. In the high strain rate region (1 s^−1^), power dissipation efficiency increases with the increase of the compression temperature. This is because volume fraction of DRX in the composite increases with the decrease of strain rate and the increase of compression temperature. When the compression temperature interval is 623 K–673 K and the strain rate range is 0.001–0.01 s^−1^, the degree of DRX is large and the distribution of grain size is even, dislocation density decreases and the degree of the stress concentration is significantly weakened in the composite. Thus, this area can be regarded as a workability region for the composite since power dissipation efficiency is high within the area. By comparing Figure 13a with Figure 13b, the AZ91 alloy and its composite have the same workability region of 600 K–673 K and 0.001–1 s^−1^, while peak dissipation efficiency of AZ91 alloy is slightly higher than that of the composite. Besides, the instability region of matrix alloy in the composite is reduced compared with that of AZ91 alloy at high temperature, indicating that the addition of SiC nanoparticles can improve the deformation ability at high temperature. At deformation condition of 523 K–573 K/0.1–1 s^−^^1^, it is difficult to activate the slip system in both the AZ91 alloy and composite, so twinning is the main deformation way in this situation. The occurrence of twins can improve deformation resistance and internal stress field, leading to the instability region. Meanwhile, compressive stress-strain curves of the AZ91 alloy and its composites indicate that flow stress at 523 K/0.1–1 s^−1^ is significantly higher than that at 523 K/0.001–0.01 s^−1^, which is consistent with the results of microstructure analysis. It can be also found that the instability region of the composite is significantly smaller than that of the AZ91 alloy under high temperature and high strain rate by comparing the processing maps of AZ91 alloy and composite. When the compression temperature is 623 K, the microstructure for the composite is mainly composed of DRX with some coarse original grains at high strain rate (0.1–1 s^−1^). With respect to AZ91 alloy, fine DRX grains are mainly distributed in the grain boundaries of the original coarse grains, forming a regional rheological structure. The stress concentration that exists in the rheological structure can produce regional rheology, resulting in flow instability.

**Figure 14:**
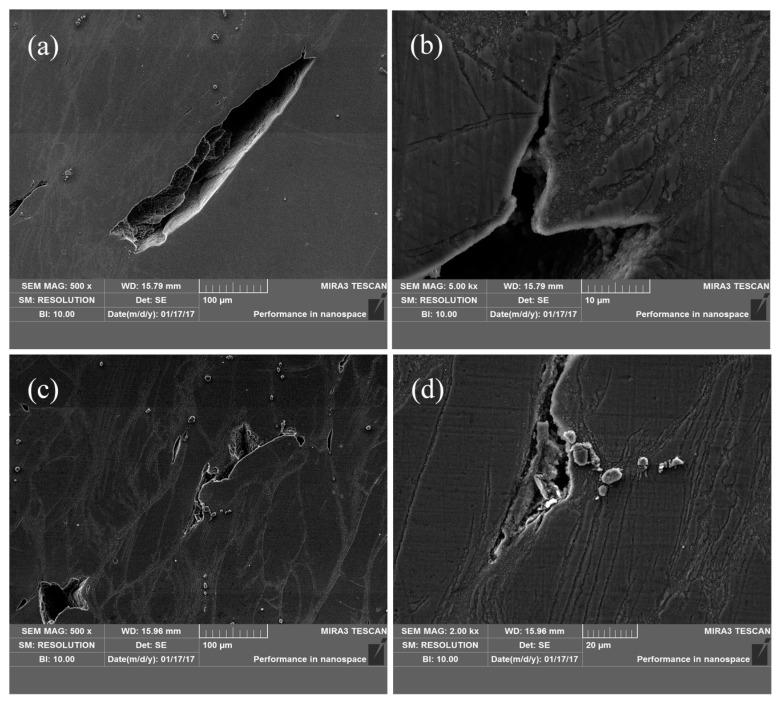
SEM images of AZ91 alloy: (**a**) 523 K and 1 s^−1^; (**c**) 523 K and 0.1 s^−1^; (**b**,**d**) high magnification of (**a**,**c**).

**Figure 15:**
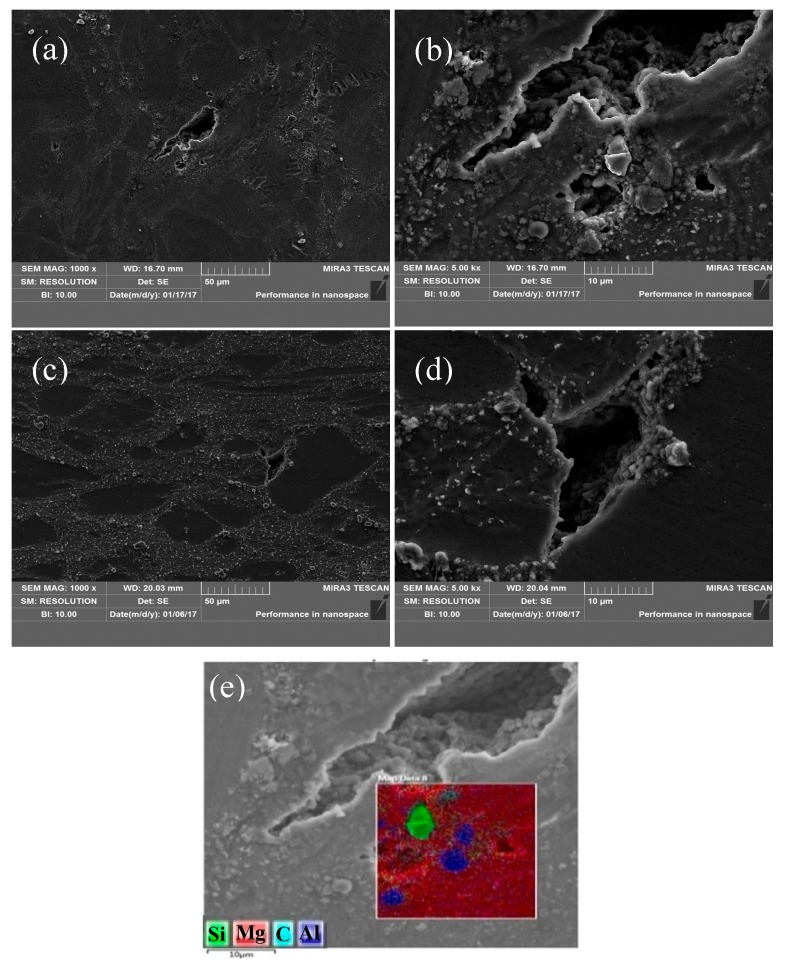
SEM images of nano-SiCp/AZ91 composite: (**a**) 523 K and 1 s^−1^; (**c**) 573 K and 1 s^−1^; (**b**,**d**) high magnification of (**a**,**c**); (**e**) the EDS of (**b**).

Figure 14 is the SEM images of AZ91 alloy under deformation condition of 523 K/0.1–1 s^−1^. It can be seen from Figure 14 that cracks are prone to generate in the area containing a large number of streamlined structures and propagate along the grain boundary (Figure 14a). The existence of large precipitates on the grain boundary can easily promote the initiation and propagation of cracks, leading to the instability of the alloy. This phenomenon is mainly due to high stress concentration existing in the streamlined structure at low temperature and large strain rate, which provides favorable conditions for the generation of cracks. Moreover, during the compression process, precipitated phase is incompatible with the matrix alloy and dislocation density near the precipitates is high, which is beneficial to the crack growth. With respect to the nano-SiCp/AZ91 composite, as shown in Figure 15, cracks occur in the particle dense zone and propagate along the grain boundary. The surface scanning analysis shows that massive Mg_17_Al_12_ phases surrounded by a large number of nanoparticles exist in the place where the crack initiates and propagates as shown in Figure 15e. With respect to magnesium matrix composites with a good interface quality, the mismatch of thermal expansion coefficients between the particles and the matrix can be accommodated by matrix plastic deformation without cracking in the particle sparse zone. However, for the particle dense zone the cracks appear as a result of a non-accommodated sliding between the matrix and the particles. Under low temperature and high strain rate, plastic deformation occurs in the AZ91 matrix alloy during hot compression, while nanoparticles with low flow ability can strongly inhibit the dislocation motion and produce high stress surrounding, leading to the appearance of micro cracks and flow instability. As shown in Figure 15d, fine second phases Mg_17_Al_12_ with large numbers of distributed at grain boundaries at low temperature and high strain rate can still impede dislocation mobility and grain boundary movement, resulting in the generation of intergranular crack.

## 4. Conclusions

In the present work, hot compression of AZ91 alloy and nano-SiCp/AZ91 composite is carried out to investigate flow behavior and processing maps. The following conclusions are drawn:

(1) The comparison of microstructures for AZ91 alloy and nano-SiCp/AZ91 composite shows that during low-temperature compression basal slip accompanied by twinning (giving c+a strain) is the main way for incoordinate deformation, while the c+a glide mechanisms becomes the main way and twinning ceases this role at high-temperature compression.
(2) By comparing compressive stress-strain curves of AZ91 alloy and nano-SiCp/AZ91 composites, the addition of nanoparticles can improve the deformation ability of the matrix alloy under high-temperature conditions. Compared with AZ91 alloy the composite has a lower critical strain and reaches its critical strain earlier.
(3) Hyperbolic sine can be applied to describe AZ91 alloy and composite under the present work. The dominant deformation mechanism is the same for both AZ91 alloy and composite, while the hot deformation activation energy of the composite is significantly lower than that of the AZ91 alloy.
(4) The AZ91 alloy and its composite have the same workability region of 600 K–673 K and 0.001–1 s^−1^, while peak dissipation efficiency of AZ91 alloy is slightly higher than that of the composite. Besides, the instability region of the composite is reduced compared with that of AZ91 alloy at high temperature.

## Figures and Tables

**Table 1:** Grain sizes of AZ91 alloy and nano-SiCp/AZ91 composite under different conditions.

Conditions (*T*/$\dot{\varepsilon }$)	AZ91 (μm)	Nano-SiCp/AZ91 (μm)
623 K/0.01 s^−1^	18.01	14.22
623 K/0.001 s^−1^	25.68	17.12
673 K/0.01 s^−1^	19.94	15.87

**Table 2:** The *n* values of AZ91 alloy.

***T* (K)**	523	573	623	673
***n***	5.5	5.5	4.7	7.4

**Table 3:** Activation energy (kJ/mol) for AZ91 alloy after hot deformation.

	$\dot{\varepsilon }$/s^−1^	0.001	0.01	0.1	1
*T* (K)					
523		153.00	155.05	156.47	164.64
573		153.00	155.05	156.47	164.64
623		130.75	132.50	133.71	140.69
673		205.86	205.86	210.52	221.51

**Table 4:** The *n* values of nano-SiCp/AZ91 composite.

***T* (K)**	523	573	623	673
***n***	3.1	4.8	5.7	5.8

**Table 5:** Activation energy (kJ/mol) for nano-SiCp/AZ91 composite after hot deformation.

	$\dot{\varepsilon }$/s^−1^	0.001	0.01	0.1	1
*T* (K)					
523		86.00	97.05	97.05	104.74
573		136.30	153.79	173.52	165.98
623		161.97	182.76	206.21	197.25
673		164.32	185.41	209.19	209.19

**Table 6:** Deformation mechanisms of AZ91 alloy and nano-SiCp/AZ91 composite.

Materials	*n*	*Q* (kJ/mol)	Deformation Mechanisms
AZ91 alloy	5.9	229	dislocation climb resulting from lattice diffusion
nano-SiCp/AZ91	4.5	158	dislocation climb resulting from lattice diffusion
